# Supplementary material for: Coupling enzymatic activity and gating in an ancient TRPM chanzyme and its molecular evolution
Source: Nat Struct Mol Biol. 2024 May 21;31(10):1509–21. doi: 10.1038/s41594-024-01316-4 (PMC11479946; doi:10.1038/s41594-024-01316-4)
Supplement: Supplementary file 1 — Supplementary Figs. 1–4 and Table 1. [file 41594_2024_1316_MOESM1_ESM.pdf]

nature structural & molecular biology

Supplementary information https://doi.org/10.1038/s41594-024-01316-4

Coupling enzymatic activity and gating in an ancient TRPM chanzyme and its molecular evolution

In the format provided by the authors and unedited

Supplementary Information

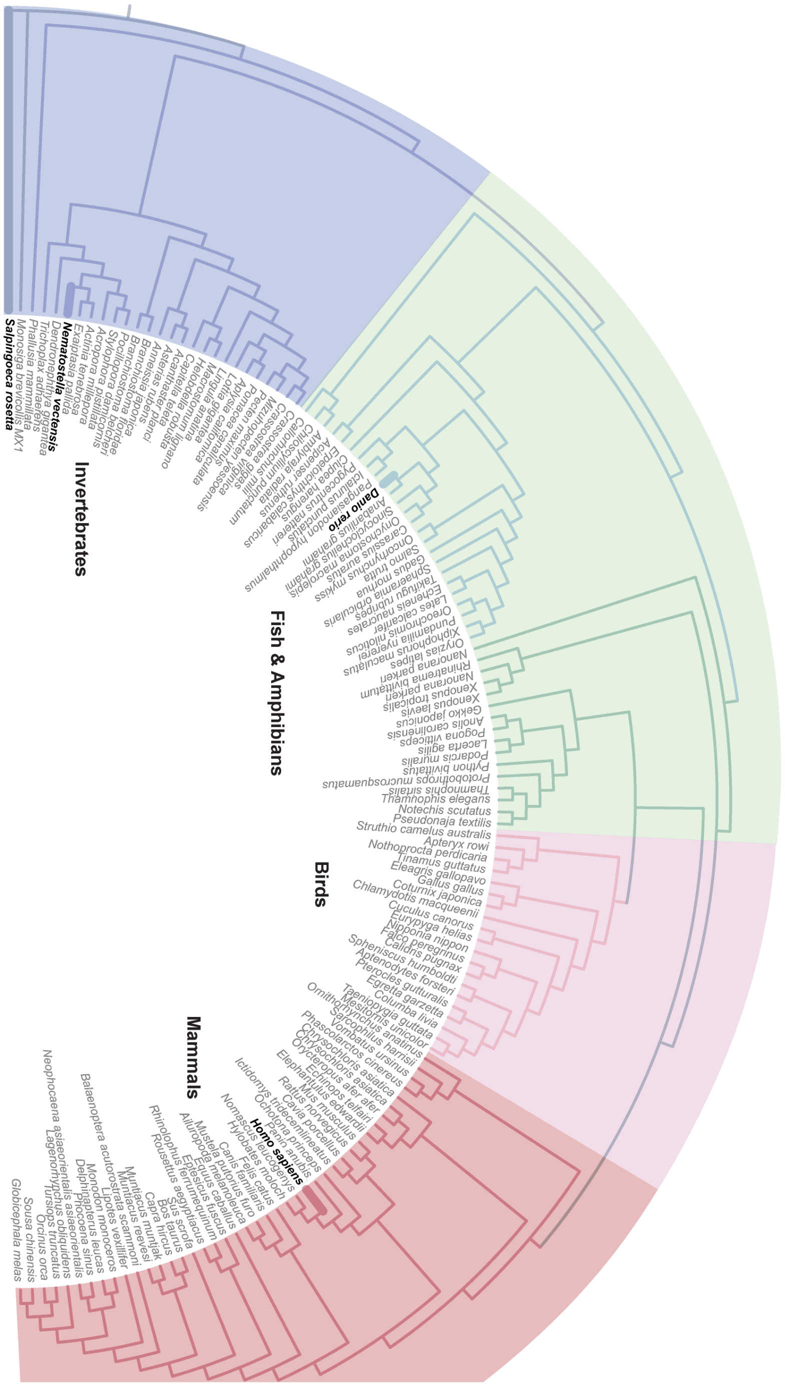

Supplementary Fig. 1 | Phylogenetic analysis of TRPM2 channels. The TRPM2 channels are divided into four groups including invertebrates, fish & amphibians, birds, and mammals. TRPM2 channels from four representative species are highlighted, including *Salpingoeca rosetta*, *Nematostella vectensis*, *Danio rerio*, and *Homo sapiens*.

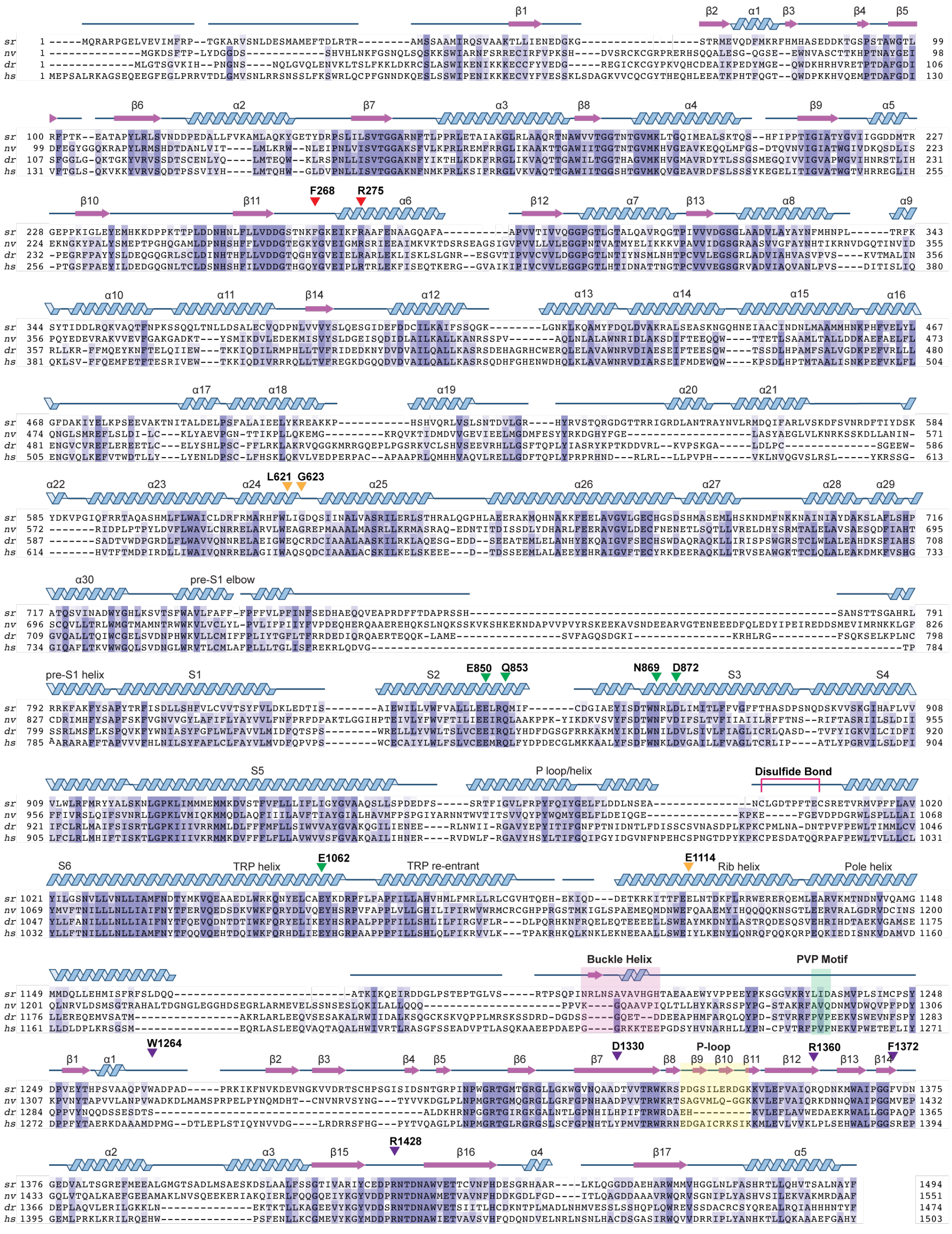

Supplementary Fig. 2 | Sequence alignment of TRPM2 from *Salpingoeca rosetta* (*sr*), *Nematostella vectensis* (*nv*), zebrafish (*dr*), human (*hs*). Secondary structures, key residues involved in ligand binding, and protein-protein interactions are indicated.

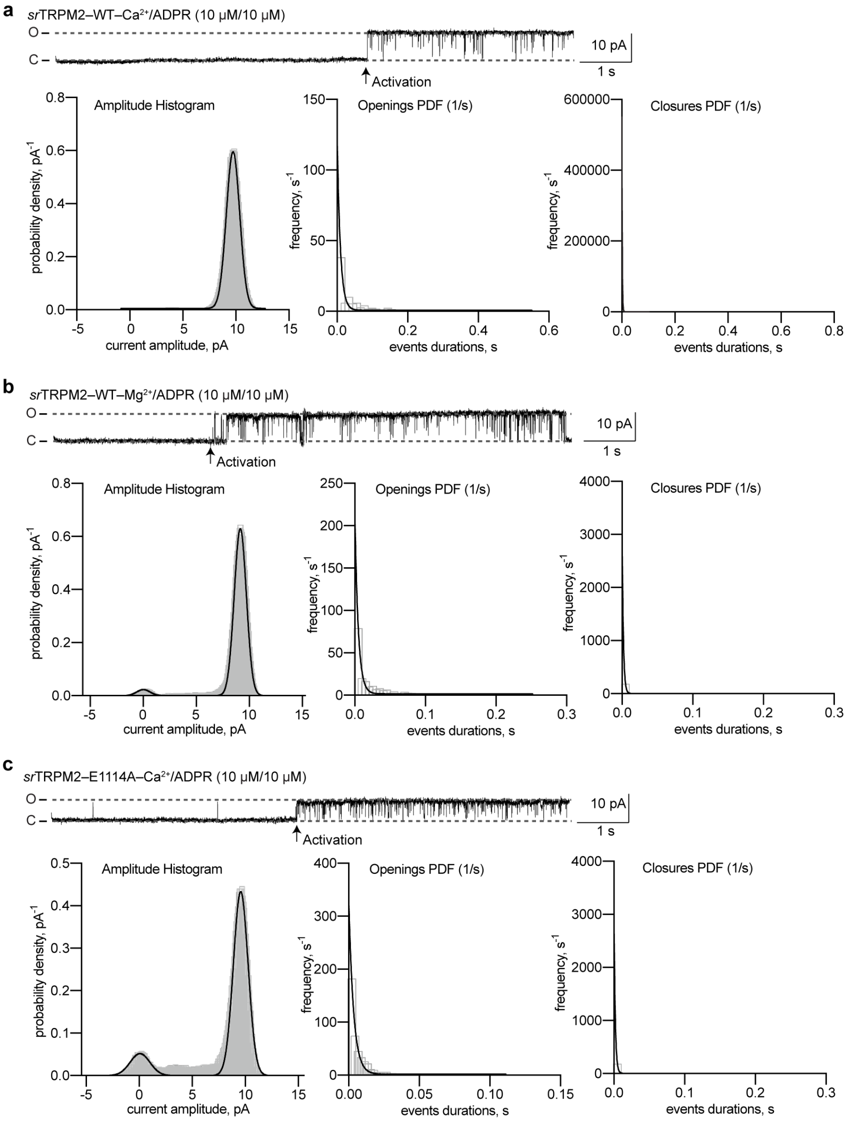

Supplementary Fig. 3 | Single channel measurments and kinetic analysis of *sr*TRPM2–WT and *sr*TRPM2–E1114A. a-c, Single channel identification with the activation point indicated and kinetics analysis including amplitude histogram, openings probability density function (PDF), and closures PDF of representative recordings. **a,** *sr*TRPM2–WT activated by 10 μM $CaCl_2$ and 10 μm ADPR. **b,** *sr*TRPM2–WT activated by 10 μM $MgCl_2$ and 10 μm ADPR. **c,** *sr*TRPM2–E1114A activated by 10 μM $CaCl_2$ and 10 μm ADPR.

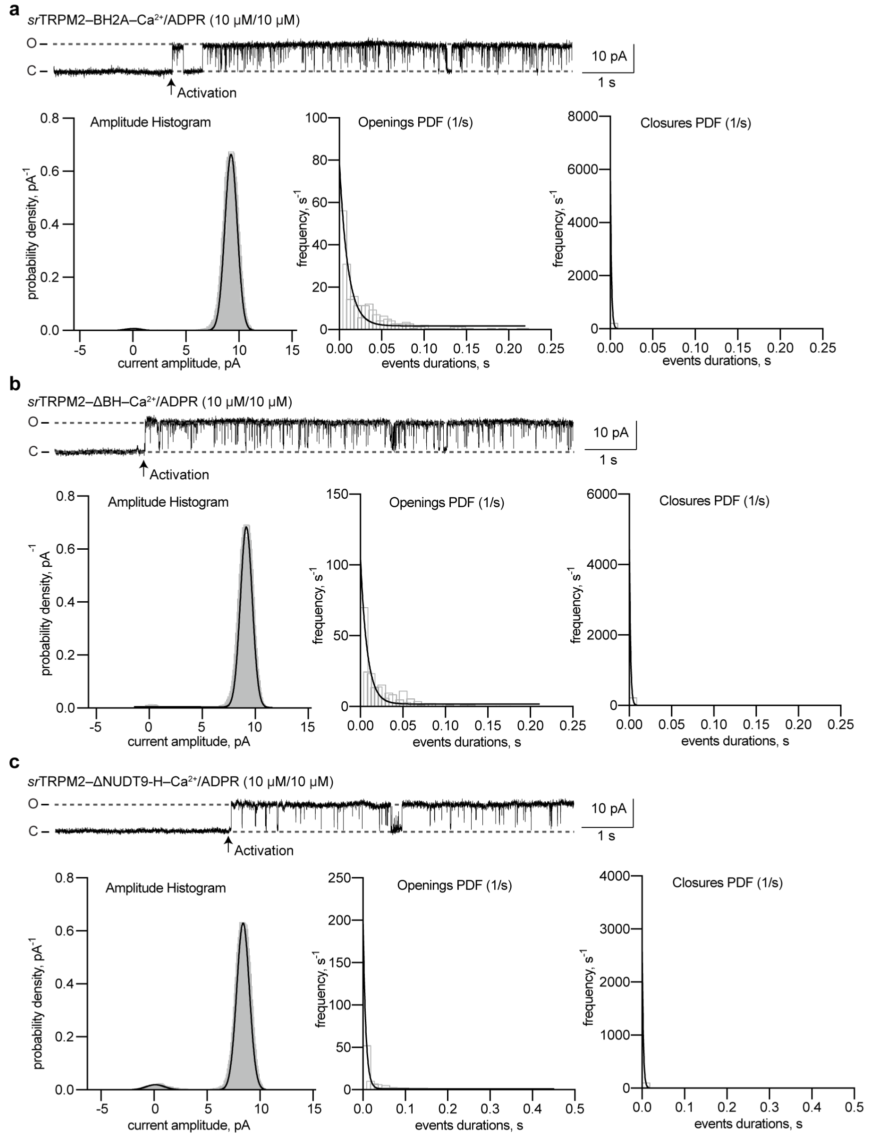

Supplementary Fig. 4 | Single channel measurments and kinetic analysis of *sr*TRPM2–BH2A, *sr*TRPM2–ΔBH, and *sr*TRPM2–ΔNUDT9-H. a-c, Single channel identification with the activation point indicated and kinetics analysis including amplitude histogram, openings probability density function (PDF), and closures PDF of representative recordings. **a,** *sr*TRPM2–BH2A activated by 10 μM $CaCl_2$ and 10 μm ADPR. **b,** *sr*TRPM2–ΔBH activated by 10 μM $CaCl_2$ and 10 μm ADPR. **c,** *sr*TRPM2–ΔNUDT9-H activated by 10 μM $CaCl_2$ and 10 μm ADPR.

Name	Sequence
F268A_Fwd	GATGGCAGCACCAATAAGGCCGGCAAGGAGATCAAGTTTA
F268A_Rev	TAAACTTGATCTCCTTGCCGGCCTTATTGGTGCTGCCATC
R275A_Fwd	GGCAAGGAGATCAAGTTTGCAGCCGCCTTCGAGAACGCAG
R275A_Rev	CTGCGTTCTCGAAGGCGGCTGCAAACTTGATCTCCTTGCC
E1114A_Fwd	CAAGATCACCACATTTGAGGCGCTGAACACAGACAAGTTC
E1114A_Rev	GAACTTGTCTGTGTTCAGCGCCTCAAATGTGGTGATCTTG
N1201C_Fwd	CCCTCTCAGCCTATCAATCGGCTGTGCAGCGCCGTGGCAGTGCACGGACAC
N1201C_Rev	GTGTCCGTGCACTGCCACGGCGCTGCACAGCCGATTGATAGGCTGAGAGGG
A1203C_Fwd	CAGCCTATCAATCGGCTGAACAGCTGCGTGGCAGTGCACGGACACACCGCA
A1203C_Rev	TGCGGTGTGTCCGTGCACTGCCACGCAGCTGTTCAGCCGATTGATAGGCTG
A1205C_Fwd	ATCAATCGGCTGAACAGCGCCGTGTGTGTGCACGGACACACCGCAGAGGCC
A1205C_Rev	GGCCTCTGCGGTGTGTCCGTGCACACACACGGCGCTGTTCAGCCGATTGAT
V1206C_Fwd	ATCAATCGGCTGAACAGCGCCGTGTGTGTGCACGGACACACCGCAGAGGCC
V1206C_Rev	GGCCTCTGCGGTGTGTCCGTGCACACACACGGCGCTGTTCAGCCGATTGAT
G1384C_Fwd	GGCGAGGATGTGGCCCTGACATCCTGCAGAGAGTTCATGGAGGAGGCCCTG
G1384C_Rev	CAGGGCCTCCTCCATGAACTCTCTGCAGGATGTCAGGGCCACATCCTCGCC
A1410C_Fwd	TCTGCCGAGAGCAAGGATTCCCTGTGCGCCCTGTTTTCCTCTGGCACAATC
A1410C_Rev	GATTGTGCCAGAGGAAAACAGGGCGCACAGGGAATCCTTGCTCTCGGCAGA
S1414C_Fwd	AAGGATTCCCTGGCCGCCCTGTTTTGTTCTGGCACAATCGTGGCCAGAATC
S1414C_Rev	GATTCTGGCCACGATTGTGCCAGAACAAAACAGGGCGGCCAGGGAATCCTT

Supplementary Table 1 | Mutagenesis primers.
