# Supplementary material for: Coupling enzymatic activity and gating in an ancient TRPM chanzyme and its molecular evolution
Source: Nat Struct Mol Biol. 2024 May 21;31(10):1509–21. doi: 10.1038/s41594-024-01316-4 (PMC11479946; doi:10.1038/s41594-024-01316-4)
Supplement: Supplementary file 2 — Reporting Summary [file 41594_2024_1316_MOESM2_ESM.pdf]

nature portfolio

Corresponding author(s): Wei Lü, Juan Du

Last updated by author(s): Feb 18, 2024

Reporting Summary

Nature Portfolio wishes to improve the reproducibility of the work that we publish. This form provides structure for consistency and transparency in reporting. For further information on Nature Portfolio policies, see our Editorial Policies and the Editorial Policy Checklist.

Statistics

For all statistical analyses, confirm that the following items are present in the figure legend, table legend, main text, or Methods section.

n/a	Confirmed	
☐	☒	The exact sample size (*n*) for each experimental group/condition, given as a discrete number and unit of measurement
☐	☒	A statement on whether measurements were taken from distinct samples or whether the same sample was measured repeatedly
☐	☒	The statistical test(s) used AND whether they are one- or two-sided<br>*Only common tests should be described solely by name; describe more complex techniques in the Methods section.*
☒	☐	A description of all covariates tested
☒	☐	A description of any assumptions or corrections, such as tests of normality and adjustment for multiple comparisons
☐	☒	A full description of the statistical parameters including central tendency (e.g. means) or other basic estimates (e.g. regression coefficient) AND variation (e.g. standard deviation) or associated estimates of uncertainty (e.g. confidence intervals)
☐	☒	For null hypothesis testing, the test statistic (e.g. *F*, *t*, *r*) with confidence intervals, effect sizes, degrees of freedom and *P* value noted<br>*Give P values as exact values whenever suitable.*
☒	☐	For Bayesian analysis, information on the choice of priors and Markov chain Monte Carlo settings
☒	☐	For hierarchical and complex designs, identification of the appropriate level for tests and full reporting of outcomes
☒	☐	Estimates of effect sizes (e.g. Cohen's *d*, Pearson's *r*), indicating how they were calculated

Our web collection on statistics for biologists contains articles on many of the points above.

Software and code

Policy information about availability of computer code

Data collection	SerialEM 3.7, Patchmaster 2x90.5
Data analysis	Gctf-1.06, Gautomatch-0.56, Relion-3.1, Relion-4.0, MolProbity4.4, CryoSparc-v3.0, MotionCorr2-1.4.0, topaz v0.2.4, Phenix v1.19.1-4122, Coot-0.9.8.1, UCSF chimera_1.13.1, UCSF chimeraX_0.91, PyMol v2.5, AlphaFold v2.1.2, GraphPad Prism 7, Nest-o-Patch 2.1.

For manuscripts utilizing custom algorithms or software that are central to the research but not yet described in published literature, software must be made available to editors and reviewers. We strongly encourage code deposition in a community repository (e.g. GitHub). See the Nature Portfolio guidelines for submitting code & software for further information.

Data

Policy information about availability of data

All manuscripts must include a data availability statement. This statement should provide the following information, where applicable:

- Accession codes, unique identifiers, or web links for publicly available datasets
- A description of any restrictions on data availability
- For clinical datasets or third party data, please ensure that the statement adheres to our policy

The cryoEM density maps and coordinates of srTRPM2–WT–apo, srTRPM2–WT–Ca2+, srTRPM2–WT–Mg2+, srTRPM2–WT–ADPR, srTRPM2–WT–Ca2+/ADPR, srTRPM2–WT–Mg2+/AMP/R5P, srTRPM2–WT–Mg2+/ADPR/4m, srTRPM2–WT–Mg2+/ADPR/10s_open, srTRPM2–WT–Mg2+/ADPR/10s_closed_intact, srTRPM2–WT–Mg2+/ADPR/10s_closed_hydrolyzed, srTRPM2–E1114A–Mg2+/ADPR/5s_open, srTRPM2–E1114A–Mg2+/ADPR/5s_closed, srTRPM2–ΔNUDT9H–apo, srTRPM2–

ΔNUDT9H–Ca2+-ADPR were deposited in the EMDB (Electron Microscopy Data Bank) under the accession numbers EMD-40722, EMD-40724, EMD-40723, EMD-40725, EMD-40726, EMD-40721, EMD-40730, EMD-40727, EMD-40728, EMD-40729, EMD-40731, EMD-40732, EMD-40733, and EMD-40734 respectively. Atomic models for srTRPM2–WT–apo, srTRPM2–WT–Ca2+, srTRPM2–WT–Mg2+, srTRPM2–WT–ADPR, srTRPM2–WT–Ca2+/ADPR, srTRPM2–WT–Mg2+/AMP/R5P, srTRPM2–WT–Mg2+/ADPR/4m, srTRPM2–WT–Mg2+/ADPR/10s_open, srTRPM2–WT–Mg2+/ADPR/10s_closed_intact, srTRPM2–WT–Mg2+/ADPR/10s_closed_hydrolyzed, srTRPM2–E1114A–Mg2+/ADPR/5s_open, srTRPM2–E1114A–Mg2+/ADPR/5s_closed, srTRPM2–ΔNUDT9H–apo, srTRPM2–ΔNUDT9H–Ca2+-ADPR were deposited in the RCSB PDB (Research Collaboratory for Structural Bioinformatics Protein Data Bank) under accession codes 8SR8, 8SRA, 8SR9, 8SRB, 8SRC, 8SR7, 8SRG, 8SRD, 8SRE, 8SRF, 8SRH, 8SRI, 8SRJ, and 8SRK respectively.

Research involving human participants, their data, or biological material

Policy information about studies with human participants or human data. See also policy information about sex, gender (identity/presentation), and sexual orientation and race, ethnicity and racism.

Reporting on sex and gender	N/A
Reporting on race, ethnicity, or other socially relevant groupings	N/A
Population characteristics	N/A
Recruitment	N/A
Ethics oversight	N/A

Note that full information on the approval of the study protocol must also be provided in the manuscript.

Field-specific reporting

Please select the one below that is the best fit for your research. If you are not sure, read the appropriate sections before making your selection.

☒ Life sciences ☐ Behavioural & social sciences ☐ Ecological, evolutionary & environmental sciences

For a reference copy of the document with all sections, see nature.com/documents/nr-reporting-summary-flat.pdf

Life sciences study design

All studies must disclose on these points even when the disclosure is negative.

Sample size	Sample sizes were determined based on preliminary experiments and prior experiences of the investigators. Cryo-EM data has been collected in sufficient quantity to enable reconstruction at a resolution of 4.2 Å or higher. Macroscopic electrophysiology measurements were performed on at least three different cells and single channel measurements were performed on at least six different cells.
Data exclusions	No data were excluded from analysis
Replication	The number of biologically independent experimental replications is indicated in the figure legend.
Randomization	In cryo-EM data processing, particles were randomly allocated to even and odd groups during refinement and resolution estimation using gold-standard Fourier shell correlation (FSC). In electrophysiology experiments, there was no predetermined order of experimentation and the cells used for patching are selected randomly.
Blinding	The investigators were not blinded when performing the experiment as it is important to use channels as a control for monitoring factors such as transfection efficiency and cell health, which could influence measurements.

Reporting for specific materials, systems and methods

We require information from authors about some types of materials, experimental systems and methods used in many studies. Here, indicate whether each material, system or method listed is relevant to your study. If you are not sure if a list item applies to your research, read the appropriate section before selecting a response.

Materials & experimental systems

n/a	Involved in the study
☒	☐ Antibodies
☐	☒ Eukaryotic cell lines
☒	☐ Palaeontology and archaeology
☒	☐ Animals and other organisms
☒	☐ Clinical data
☒	☐ Dual use research of concern
☒	☐ Plants

Methods

n/a	Involved in the study
☒	☐ ChIP-seq
☒	☐ Flow cytometry
☒	☐ MRI-based neuroimaging

Eukaryotic cell lines

Policy information about cell lines and Sex and Gender in Research

Cell line source(s)	Sf9 cells and tsA201 cells were purchased from ATCC.
Authentication	These cells were purchased and routinely maintained in our lab, and were not authenticated experimentally in this study.
Mycoplasma contamination	sf9 cells and tsA201 cells were tested negative for Mycoplasma contamination.
Commonly misidentified lines (See ICLAC register)	No commonly misidentified cell lines were used.

Plants

Seed stocks	*Report on the source of all seed stocks or other plant material used. If applicable, state the seed stock centre and catalogue number. If plant specimens were collected from the field, describe the collection location, date and sampling procedures.*
Novel plant genotypes	*Describe the methods by which all novel plant genotypes were produced. This includes those generated by transgenic approaches, gene editing, chemical/radiation-based mutagenesis and hybridization. For transgenic lines, describe the transformation method, the number of independent lines analyzed and the generation upon which experiments were performed. For gene-edited lines, describe the editor used, the endogenous sequence targeted for editing, the targeting guide RNA sequence (if applicable) and how the editor was applied.*
Authentication	*Describe any authentication procedures for each seed stock used or novel genotype generated. Describe any experiments used to assess the effect of a mutation and, where applicable, how potential secondary effects (e.g. second site T-DNA insertions, mosiacism, off-target gene editing) were examined.*
