# Supplementary material for: Coupling enzymatic activity and gating in an ancient TRPM chanzyme and its molecular evolution
Source: Nat Struct Mol Biol. 2024 May 21;31(10):1509–21. doi: 10.1038/s41594-024-01316-4 (PMC11479946; doi:10.1038/s41594-024-01316-4)

*sr*TRPM2–WT–apo

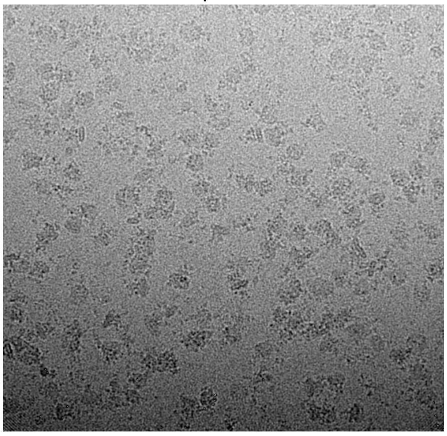

*sr*TRPM2–WT–Ca^{2+}

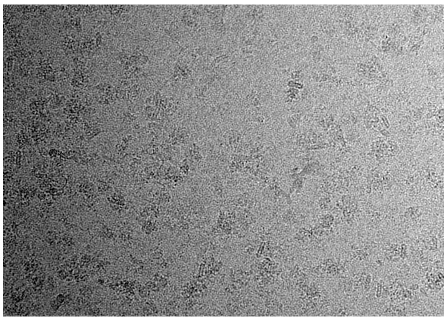

*sr*TRPM2–WT–Mg^{2+}

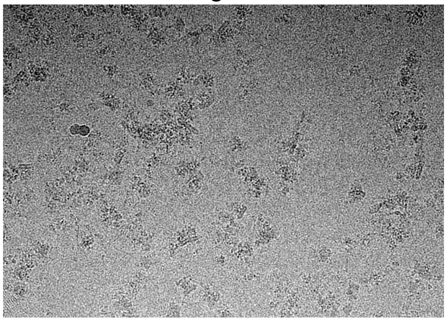

*sr*TRPM2–WT–ADPR

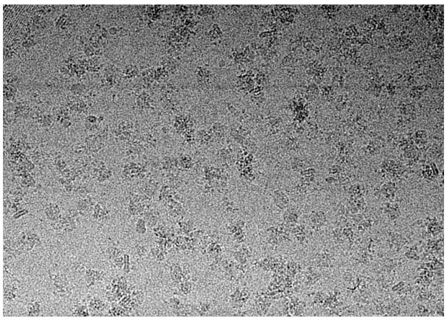

*sr*TRPM2–WT–Ca^{2+}/ADPR

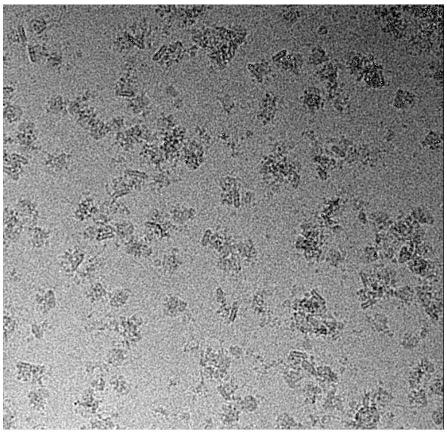

*sr*TRPM2–WT–Mg^{2+}/AMP/R5P

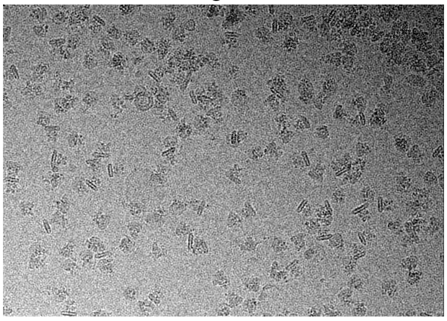

*sr*TRPM2–WT–Mg^{2+}/ADPR/4m

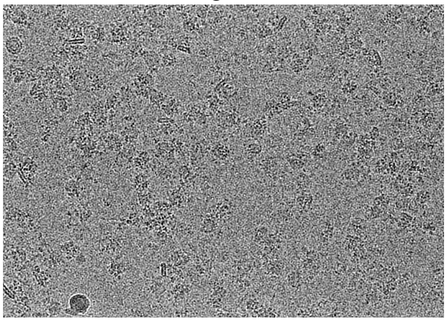

*sr*TRPM2–WT–Mg^{2+}/ADPR/10s

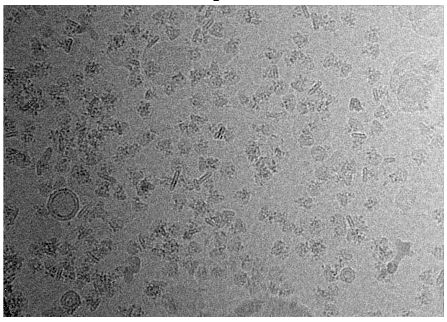

*sr*TRPM2–E1114A–Mg^{2+}/ADPR/5s

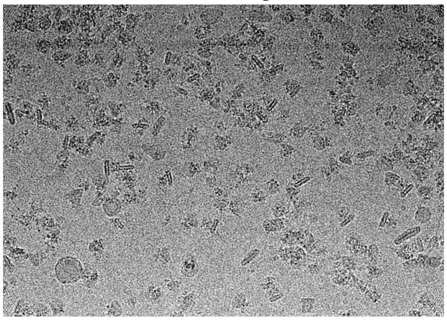

*sr*TRPM2–ΔNUDT9H–apo

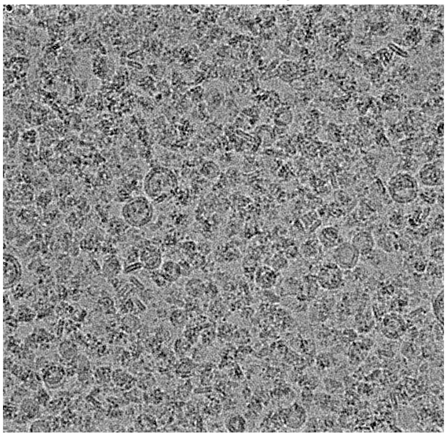

*srT*RPM2–ΔNUDT9H–Ca^{2+}/ADPR

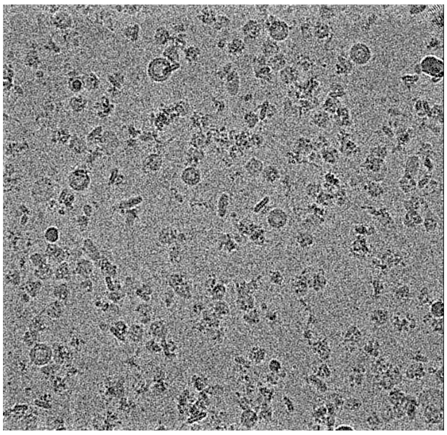

Supplement: Supplementary file 5 — Representative micrographs of the cryo-EM datasets. [file 41594_2024_1316_MOESM5_ESM.pdf]
