# Supplementary figures and images for: Coupling enzymatic activity and gating in an ancient TRPM chanzyme and its molecular evolution
Source: Nat Struct Mol Biol. 2024 May 21;31(10):1509–21. doi: 10.1038/s41594-024-01316-4 (PMC11479946; doi:10.1038/s41594-024-01316-4)

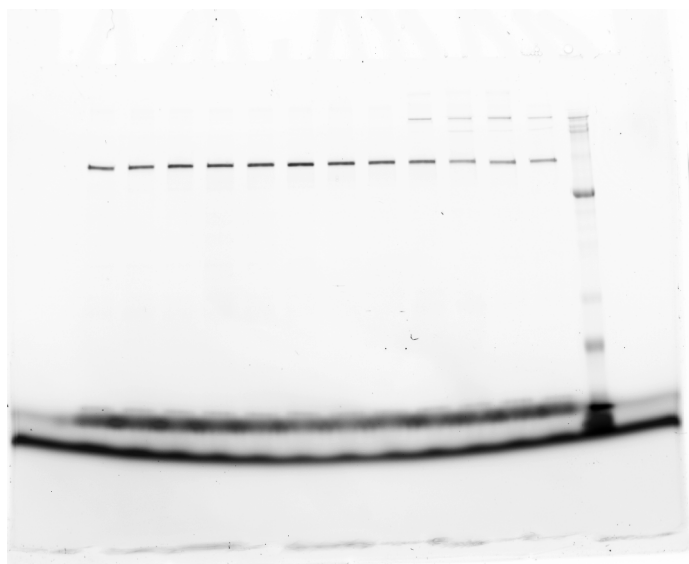

Supplement: Supplementary file 6 — Unprocessed gels. [file 41594_2024_1316_MOESM6_ESM.pdf]

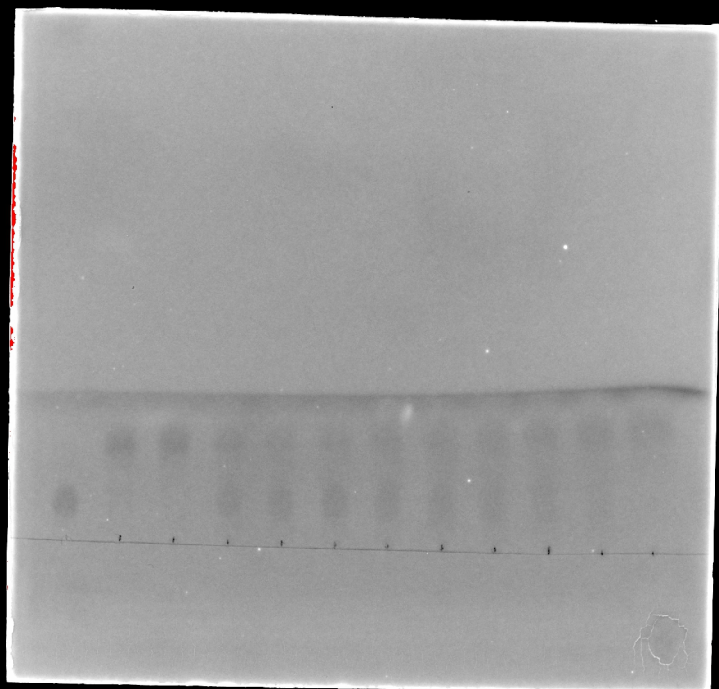

Supplement: Supplementary file 9 — Unprocessed thin-layer chromatography image. [file 41594_2024_1316_MOESM9_ESM.pdf]
